# 1-[4-(3-{[5-(4-Chloro­phen­yl)furan-2-yl]methyl­idene­amino}-2,5-dioxoimidazol­idin-1-yl)but­yl]-4-methyl­piperazine-1,4-diium dichloride hemihydrate

**DOI:** 10.1107/S1600536811000560

**Published:** 2011-01-12

**Authors:** Yan-Shu Liang, Shuai Mu, Ying Liu, Deng-Ke Liu

**Affiliations:** a Tianjin University of Commerce, Tianjin 300134, People’s Republic of China; b School of Chemical Engineering and Technology, Tianjin University, Tianjin, 300072, People’s Republic of China; c Tianjin Institute of Pharmaceutical Research, Tianjin, 300193, People’s Republic of China

## Abstract

The title compound, C_23_H_30_ClN_5_O_3_^2+^·2Cl^−^·0.5H_2_O, was synthesized by *N*-alkyl­ation of 1-({[5-(4-chloro­phen­yl)-2-furan­yl]methyl­ene}amino)-2,4-imidazolidinedione with 1-bromo-4-chloro­butane, and *N*-methyl­piperazine. In the crystal, the cations, anions and water mol­ecules are linked by O—H⋯Cl and N—H⋯Cl hydrogen bonds.

## Related literature

For bond-length data, see: Allen *et al.* (1987 ▶). For background to the bioactivity and applications of the title compound, see: Pratt *et al.* (2004 ▶). For the preparation of the title compound, see: Matson *et al.* (1999 ▶).
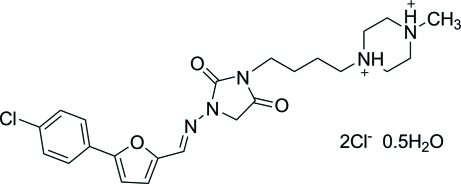


## Experimental

### 

#### Crystal data

C_23_H_30_ClN_5_O_3_^2+^·2Cl^−^·0.5H_2_O
*M* *_r_* = 539.88
Monoclinic, 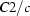

*a* = 59.563 (12) Å
*b* = 6.8793 (14) Å
*c* = 12.831 (2) Å
β = 94.402 (4)°
*V* = 5241.9 (18) Å^3^
*Z* = 8
Mo *K*α radiation
μ = 0.39 mm^−1^
*T* = 294 K
0.20 × 0.14 × 0.08 mm

#### Data collection

Bruker SMART CCD area-detector diffractometer
Absorption correction: multi-scan (*SADABS*; Sheldrick, 1996 ▶) *T*_min_ = 0.725, *T*_max_ = 1.000
12667 measured reflections
4629 independent reflections
2498 reflections with *I* > 2σ(*I*)
*R*_int_ = 0.059

#### Refinement

*R*[*F* ^2^ > 2σ(*F* ^2^)] = 0.045
*wR*(*F* ^2^) = 0.123
*S* = 1.00
4629 reflections
321 parameters
H atoms treated by a mixture of independent and constrained refinement
Δρ_max_ = 0.34 e Å^−3^
Δρ_min_ = −0.28 e Å^−3^

### 

Data collection: *SMART* (Bruker, 1997 ▶); cell refinement: *SAINT* (Bruker, 1997 ▶); data reduction: *SAINT*; program(s) used to solve structure: *SHELXS97* (Sheldrick, 2008 ▶); program(s) used to refine structure: *SHELXL97* (Sheldrick, 2008 ▶); molecular graphics: *SHELXTL* (Sheldrick, 2008 ▶); software used to prepare material for publication: *SHELXTL*.

## Supplementary Material

Crystal structure: contains datablocks global, I. DOI: 10.1107/S1600536811000560/kp2297sup1.cif

Structure factors: contains datablocks I. DOI: 10.1107/S1600536811000560/kp2297Isup2.hkl

Additional supplementary materials:  crystallographic information; 3D view; checkCIF report

## Figures and Tables

**Table 1:** Hydrogen-bond geometry (Å, °)

*D*—H⋯*A*	*D*—H	H⋯*A*	*D*⋯*A*	*D*—H⋯*A*
O4—H4*B*⋯Cl3	0.86	2.40	3.250 (3)	169
O4—H4*C*⋯Cl3^i^	0.85	2.50	3.250 (3)	148
N5—H5*A*⋯Cl2^ii^	1.05 (4)	1.95 (4)	2.995 (3)	174 (3)
N4—H4*A*⋯Cl3	1.04 (4)	2.00 (4)	3.035 (3)	178 (3)

Symmetry codes: (i) 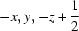; (ii) 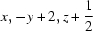.

**Fig. 1.**
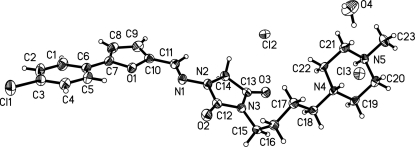
The molecular structure of (I) with the atom-numbering scheme and 30% probability displacement ellipsoids.

**Fig. 2.**
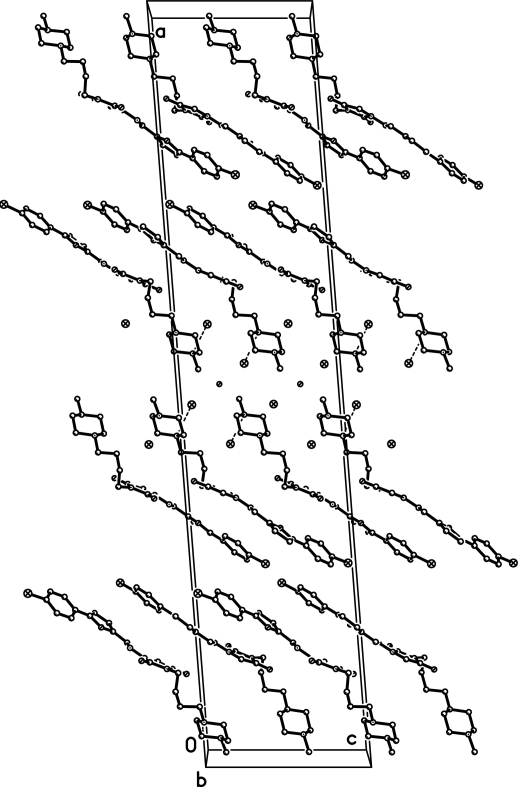
Packing diagram for (I) with hydrogen bonds drawn by dashed lines.

## supplementary crystallographic information

### Comment

The compound, 1-[[[5-(4-chlorophenyl)-2-furanyl]methylene]amino]-3- [4-(4-methyl-1-piperazinyl)butyl]-2, 4-imidazolidinedione dihydrochloride (Azimilide dihydrochloride), (I), is a Vaughan-Williamsclass III antiarrhythmic drug, which is being developed primarily for atrial fibrillation and also as adjunctive antiarrhythmic therapy in patients with implantable cardioverter defibrillators (Pratt *et al.*, 2004). Now, we present the crystal structure of the title compound (Fig. 1).

All bond lengths and angles in (I) are within normal ranges (Allen *et al.*, 1987) except for the N—H bonds involved in hydrogen bonds with charged donors N4—H4A (1.04 Å) and N5—H5A (1.05 Å)of piperazonia ring, and also chloride ions Cl_2_^-^ and Cl_3_^-^ as the acceptors. In the crystal, the imidazolidine-2,4-dione ring (C12/C13/C14/N2/N3/O2/O3) is planar with the r.m.s. deviation of 0.0029 Å. The dihedral angle formed between the imidazolidine-2,4-dione ring plane (A), the benzene ring plane (C1 to C6, B) and the furan ring plane (C7/C8/C9/C10/O1, C) are 28.67 (15) ° (A/B), 12.06 (3) ° (B/C) and 18.73 (18) ° (A/C), respectively. The piperazonia ring adopts a chair conformation. The packing is stabilized by intermolecular O—H···Cl and N—H···Cl hydrogen bonds (Table 1 and Fig. 2).

### Experimental

The title compound was prepared according to the method of Matson *et al.* (1999). A three-neck 1 *L* flask fitted with a thermometer, mechanical stirrer, heating mantle, reflux condenser and addition funnel is charged with DMF (240 ml) and heated to 323 K. 1-[[[5-(4-Chlorophenyl)-2-furanyl]methylene]amino]-2,4- imidazolidinedione (29 g) is added and heating is continued. When dissolution is completed, potassium carbonate (14 g) is charged to the flask and heating is continued to 353 K. After 20 minutes, 1-bromo-4-chlorobutane (18.5 g) is added, and heating is continued to approximately 373 K. After 50 minutes, *N*-methylpiperazine (23.5 g) is added, and the mixture is allowed to stir for 2 h at 373 K. The reaction mixture is cooled to approximately 283 K and filtered to remove insolubles. The DMF is removed under reduced pressure at 338–341 K and replaced with absolute ethanol (175 ml). The mixture is heated to dissolve the free base and filtered to remove insolubles. The product is precipitated from ethanol (300 ml total) with the addition of 20 g of concentrated hydrochloric acid and then filtered to give 31 g of Azimilide dihydrochloride. The above dihydrochloride is suspended in 670 ml of refluxing ethanol and 150 ml of water are added to obtain complete solubilization. The mixture was standing under 298 K, then white crystals were grown slowly. The crystals were washed with cold ethanol, yield 25.6 g.

### Refinement

The two water H atoms were located in a difference Fourier map and then refined as riding on the water O atom (0.85 and 0.86 Å). Other H atoms were positioned geometrically and refined using a riding model, with d(C—H) = 0.93 - 0.97 Å and *U*_iso_(H) = 1.2*U*_eq_(C) or 1.5*U*_eq_(C_methyl_).

### Crystal data

**Table:**

C_23_H_30_ClN_5_O_3_^2+^·2Cl^−^·0.5H_2_O	*F*(000) = 2264
*M**_r_* = 539.88	*D*_x_ = 1.368 Mg m^−^^3^
Monoclinic, *C*2/*c*	Mo *K*α radiation, λ = 0.71073 Å
Hall symbol: -C 2yc	Cell parameters from 2226 reflections
*a* = 59.563 (12) Å	θ = 2.7–27.7°
*b* = 6.8793 (14) Å	µ = 0.39 mm^−^^1^
*c* = 12.831 (2) Å	*T* = 294 K
β = 94.402 (4)°	Prism, white
*V* = 5241.9 (18) Å^3^	0.20 × 0.14 × 0.08 mm
*Z* = 8	

### Data collection

**Table:**

Bruker SMART CCD area-detector diffractometer	4629 independent reflections
Radiation source: fine-focus sealed tube	2498 reflections with *I* > 2σ(*I*)
graphite	*R*_int_ = 0.059
φ and ω scans	θ_max_ = 25.0°, θ_min_ = 0.7°
Absorption correction: multi-scan (*SADABS*; Sheldrick, 1996)	*h* = −70→70
*T*_min_ = 0.725, *T*_max_ = 1.000	*k* = −3→8
12667 measured reflections	*l* = −14→15

### Refinement

**Table:**

Refinement on *F*^2^	Primary atom site location: structure-invariant direct methods
Least-squares matrix: full	Secondary atom site location: difference Fourier map
*R*[*F*^2^ > 2σ(*F*^2^)] = 0.045	Hydrogen site location: inferred from neighbouring sites
*wR*(*F*^2^) = 0.123	H atoms treated by a mixture of independent and constrained refinement
*S* = 1.00	*w* = 1/[σ^2^(*F*_o_^2^) + (0.0367*P*)^2^ + 5.8309*P*] where *P* = (*F*_o_^2^ + 2*F*_c_^2^)/3
4629 reflections	(Δ/σ)_max_ = 0.002
321 parameters	Δρ_max_ = 0.34 e Å^−^^3^
0 restraints	Δρ_min_ = −0.28 e Å^−^^3^

### Special details

**Table:**

Geometry. All e.s.d.'s (except the e.s.d. in the dihedral angle between two l.s. planes) are estimated using the full covariance matrix. The cell e.s.d.'s are taken into account individually in the estimation of e.s.d.'s in distances, angles and torsion angles; correlations between e.s.d.'s in cell parameters are only used when they are defined by crystal symmetry. An approximate (isotropic) treatment of cell e.s.d.'s is used for estimating e.s.d.'s involving l.s. planes.
Refinement. Refinement of *F*^2^ against ALL reflections. The weighted *R*-factor *wR* and goodness of fit *S* are based on *F*^2^, conventional *R*-factors *R* are based on *F*, with *F* set to zero for negative *F*^2^. The threshold expression of *F*^2^ > σ(*F*^2^) is used only for calculating *R*-factors(gt) *etc*. and is not relevant to the choice of reflections for refinement. *R*-factors based on *F*^2^ are statistically about twice as large as those based on *F*, and *R*- factors based on ALL data will be even larger.

### Fractional atomic coordinates and isotropic or equivalent isotropic displacement parameters (Å^2^)

**Table:**

	*x*	*y*	*z*	*U*_iso_*/*U*_eq_	Occ. (<1)
Cl1	0.23396 (2)	0.4537 (2)	−0.45468 (9)	0.0911 (5)	
O1	0.18447 (4)	0.8711 (4)	−0.05974 (19)	0.0462 (7)	
O2	0.14878 (5)	0.4220 (4)	0.1843 (2)	0.0589 (8)	
O3	0.12471 (4)	0.8338 (4)	0.43121 (19)	0.0515 (7)	
N1	0.15913 (5)	0.7902 (5)	0.1069 (2)	0.0440 (8)	
N2	0.14652 (5)	0.7590 (5)	0.1904 (2)	0.0448 (8)	
N3	0.13558 (5)	0.5894 (4)	0.3246 (2)	0.0400 (8)	
N4	0.06729 (5)	0.5843 (4)	0.5197 (2)	0.0337 (7)	
N5	0.04067 (5)	0.8887 (5)	0.6061 (2)	0.0390 (8)	
C1	0.22367 (7)	0.9011 (7)	−0.2680 (3)	0.0650 (13)	
H1	0.2290	1.0276	−0.2592	0.078*	
C2	0.23197 (7)	0.7838 (9)	−0.3432 (3)	0.0690 (14)	
H2	0.2430	0.8308	−0.3840	0.083*	
C3	0.22404 (7)	0.5989 (8)	−0.3580 (3)	0.0594 (12)	
C4	0.20785 (7)	0.5277 (7)	−0.2977 (3)	0.0630 (13)	
H4	0.2023	0.4022	−0.3083	0.076*	
C5	0.19981 (7)	0.6438 (7)	−0.2213 (3)	0.0573 (12)	
H5	0.1890	0.5946	−0.1797	0.069*	
C6	0.20750 (6)	0.8320 (7)	−0.2054 (3)	0.0485 (11)	
C7	0.19819 (7)	0.9568 (6)	−0.1273 (3)	0.0478 (11)	
C8	0.19909 (7)	1.1508 (7)	−0.1081 (3)	0.0600 (12)	
H8	0.2073	1.2418	−0.1429	0.072*	
C9	0.18542 (7)	1.1891 (7)	−0.0263 (3)	0.0574 (12)	
H9	0.1827	1.3098	0.0030	0.069*	
C10	0.17690 (7)	1.0172 (6)	0.0018 (3)	0.0452 (10)	
C11	0.16305 (6)	0.9676 (6)	0.0845 (3)	0.0444 (10)	
H11	0.1568	1.0662	0.1225	0.053*	
C12	0.14429 (6)	0.5725 (6)	0.2266 (3)	0.0439 (10)	
C13	0.13208 (6)	0.7804 (6)	0.3510 (3)	0.0394 (9)	
C14	0.13935 (6)	0.9033 (5)	0.2631 (3)	0.0422 (10)	
H14A	0.1270	0.9805	0.2321	0.051*	
H14B	0.1517	0.9888	0.2867	0.051*	
C15	0.13341 (6)	0.4226 (6)	0.3940 (3)	0.0461 (10)	
H15A	0.1346	0.4677	0.4658	0.055*	
H15B	0.1458	0.3340	0.3857	0.055*	
C16	0.11156 (6)	0.3135 (6)	0.3737 (3)	0.0464 (10)	
H16A	0.1118	0.2014	0.4197	0.056*	
H16B	0.1106	0.2658	0.3024	0.056*	
C17	0.09061 (6)	0.4316 (6)	0.3897 (3)	0.0424 (10)	
H17A	0.0774	0.3566	0.3660	0.051*	
H17B	0.0907	0.5486	0.3475	0.051*	
C18	0.08908 (6)	0.4875 (6)	0.5033 (3)	0.0413 (10)	
H18A	0.0906	0.3719	0.5464	0.050*	
H18B	0.1014	0.5747	0.5248	0.050*	
C19	0.06381 (6)	0.5970 (6)	0.6343 (2)	0.0426 (10)	
H19A	0.0643	0.4676	0.6644	0.051*	
H19B	0.0758	0.6730	0.6695	0.051*	
C20	0.04159 (6)	0.6894 (6)	0.6511 (3)	0.0448 (10)	
H20A	0.0295	0.6113	0.6179	0.054*	
H20B	0.0396	0.6956	0.7253	0.054*	
C21	0.04333 (7)	0.8767 (6)	0.4918 (3)	0.0463 (10)	
H21A	0.0426	1.0061	0.4617	0.056*	
H21B	0.0312	0.8002	0.4581	0.056*	
C22	0.06551 (6)	0.7845 (5)	0.4732 (3)	0.0413 (10)	
H22A	0.0776	0.8647	0.5041	0.050*	
H22B	0.0671	0.7767	0.3987	0.050*	
C23	0.01982 (7)	0.9928 (7)	0.6300 (3)	0.0593 (12)	
H23A	0.0195	1.1188	0.5977	0.089*	
H23B	0.0195	1.0073	0.7043	0.089*	
H23C	0.0069	0.9196	0.6034	0.089*	
Cl2	0.080376 (16)	0.89703 (15)	0.20687 (7)	0.0483 (3)	
Cl3	0.027191 (17)	0.36360 (15)	0.41881 (7)	0.0530 (3)	
H4A	0.0538 (6)	0.507 (5)	0.485 (3)	0.055 (12)*	
H5A	0.0542 (7)	0.972 (6)	0.638 (3)	0.069 (13)*	
O4	0.0000	0.6464 (6)	0.2500	0.0960 (16)	
H4C	−0.0016	0.5712	0.1973	0.115*	0.50
H4B	0.0068	0.5826	0.3011	0.115*	0.50

### Atomic displacement parameters (Å^2^)

**Table:**

	*U*^11^	*U*^22^	*U*^33^	*U*^12^	*U*^13^	*U*^23^
Cl1	0.0934 (10)	0.1185 (12)	0.0647 (8)	0.0178 (9)	0.0280 (7)	−0.0075 (8)
O1	0.0471 (16)	0.0461 (17)	0.0460 (15)	−0.0059 (14)	0.0077 (13)	0.0043 (14)
O2	0.081 (2)	0.0401 (17)	0.0583 (18)	0.0067 (16)	0.0215 (15)	−0.0113 (15)
O3	0.0632 (18)	0.0497 (18)	0.0428 (15)	0.0052 (15)	0.0115 (14)	−0.0118 (14)
N1	0.049 (2)	0.043 (2)	0.0403 (18)	−0.0018 (18)	0.0086 (16)	−0.0052 (17)
N2	0.059 (2)	0.0347 (19)	0.0426 (18)	0.0017 (18)	0.0164 (16)	−0.0054 (17)
N3	0.0466 (19)	0.0356 (19)	0.0383 (17)	0.0059 (16)	0.0072 (15)	−0.0029 (16)
N4	0.044 (2)	0.0320 (18)	0.0260 (15)	0.0009 (17)	0.0065 (14)	0.0021 (14)
N5	0.0400 (19)	0.037 (2)	0.0403 (18)	−0.0005 (17)	0.0081 (15)	−0.0068 (16)
C1	0.057 (3)	0.077 (4)	0.062 (3)	−0.017 (3)	0.011 (2)	0.005 (3)
C2	0.055 (3)	0.099 (4)	0.055 (3)	−0.012 (3)	0.021 (2)	0.003 (3)
C3	0.053 (3)	0.078 (4)	0.048 (3)	0.011 (3)	0.009 (2)	0.003 (3)
C4	0.069 (3)	0.065 (3)	0.056 (3)	0.001 (3)	0.014 (2)	0.010 (3)
C5	0.057 (3)	0.066 (3)	0.051 (3)	−0.003 (3)	0.017 (2)	0.007 (2)
C6	0.038 (2)	0.063 (3)	0.044 (2)	0.000 (2)	0.0021 (19)	0.011 (2)
C7	0.045 (3)	0.055 (3)	0.043 (2)	−0.007 (2)	0.002 (2)	0.012 (2)
C8	0.063 (3)	0.055 (3)	0.062 (3)	−0.016 (3)	0.004 (2)	0.015 (3)
C9	0.063 (3)	0.049 (3)	0.060 (3)	−0.011 (2)	0.000 (2)	0.000 (2)
C10	0.045 (3)	0.047 (3)	0.043 (2)	0.004 (2)	−0.0029 (19)	−0.003 (2)
C11	0.045 (2)	0.046 (3)	0.041 (2)	0.000 (2)	0.0013 (19)	−0.003 (2)
C12	0.042 (2)	0.044 (3)	0.046 (2)	0.003 (2)	0.0045 (19)	−0.002 (2)
C13	0.037 (2)	0.039 (2)	0.043 (2)	0.005 (2)	0.0008 (18)	−0.004 (2)
C14	0.046 (2)	0.037 (2)	0.044 (2)	0.000 (2)	0.0066 (18)	−0.008 (2)
C15	0.048 (3)	0.048 (3)	0.043 (2)	0.018 (2)	0.0067 (19)	0.008 (2)
C16	0.055 (3)	0.037 (2)	0.049 (2)	0.007 (2)	0.013 (2)	−0.002 (2)
C17	0.047 (2)	0.042 (2)	0.039 (2)	0.001 (2)	0.0049 (18)	−0.0055 (19)
C18	0.043 (2)	0.046 (2)	0.036 (2)	0.007 (2)	0.0035 (18)	0.0026 (19)
C19	0.061 (3)	0.043 (2)	0.0250 (18)	0.003 (2)	0.0079 (17)	0.0050 (18)
C20	0.056 (3)	0.045 (3)	0.035 (2)	−0.007 (2)	0.0135 (19)	−0.001 (2)
C21	0.060 (3)	0.042 (3)	0.037 (2)	0.007 (2)	0.0073 (19)	−0.001 (2)
C22	0.058 (3)	0.033 (2)	0.034 (2)	0.002 (2)	0.0128 (18)	0.0069 (18)
C23	0.049 (3)	0.066 (3)	0.064 (3)	0.008 (2)	0.017 (2)	−0.016 (3)
Cl2	0.0508 (6)	0.0441 (6)	0.0489 (6)	0.0031 (5)	−0.0043 (5)	0.0066 (5)
Cl3	0.0545 (7)	0.0547 (7)	0.0496 (6)	−0.0077 (6)	0.0021 (5)	−0.0077 (5)
O4	0.156 (5)	0.046 (3)	0.086 (3)	0.000	0.014 (3)	0.000

### Geometric parameters (Å, °)

**Table:**

Cl1—C3	1.732 (4)	C9—C10	1.347 (5)
O1—C7	1.370 (4)	C9—H9	0.9300
O1—C10	1.375 (4)	C10—C11	1.434 (5)
O2—C12	1.208 (4)	C11—H11	0.9300
O3—C13	1.207 (4)	C13—C14	1.499 (5)
N1—C11	1.279 (5)	C14—H14A	0.9700
N1—N2	1.372 (4)	C14—H14B	0.9700
N2—C12	1.374 (5)	C15—C16	1.507 (5)
N2—C14	1.449 (4)	C15—H15A	0.9700
N3—C13	1.376 (5)	C15—H15B	0.9700
N3—C12	1.402 (4)	C16—C17	1.516 (5)
N3—C15	1.464 (4)	C16—H16A	0.9700
N4—C18	1.488 (4)	C16—H16B	0.9700
N4—C22	1.501 (4)	C17—C18	1.517 (4)
N4—C19	1.504 (4)	C17—H17A	0.9700
N4—H4A	1.04 (4)	C17—H17B	0.9700
N5—C23	1.486 (4)	C18—H18A	0.9700
N5—C20	1.487 (5)	C18—H18B	0.9700
N5—C21	1.489 (4)	C19—C20	1.498 (5)
N5—H5A	1.05 (4)	C19—H19A	0.9700
C1—C2	1.378 (6)	C19—H19B	0.9700
C1—C6	1.385 (5)	C20—H20A	0.9700
C1—H1	0.9300	C20—H20B	0.9700
C2—C3	1.365 (6)	C21—C22	1.501 (5)
C2—H2	0.9300	C21—H21A	0.9700
C3—C4	1.372 (6)	C21—H21B	0.9700
C4—C5	1.378 (6)	C22—H22A	0.9700
C4—H4	0.9300	C22—H22B	0.9700
C5—C6	1.384 (6)	C23—H23A	0.9600
C5—H5	0.9300	C23—H23B	0.9600
C6—C7	1.460 (5)	C23—H23C	0.9600
C7—C8	1.357 (6)	O4—H4C	0.8500
C8—C9	1.402 (5)	O4—H4B	0.8627
C8—H8	0.9300		
			
C7—O1—C10	106.7 (3)	N2—C14—H14A	111.3
C11—N1—N2	116.5 (3)	C13—C14—H14A	111.3
N1—N2—C12	118.9 (3)	N2—C14—H14B	111.3
N1—N2—C14	127.0 (3)	C13—C14—H14B	111.3
C12—N2—C14	112.4 (3)	H14A—C14—H14B	109.2
C13—N3—C12	111.9 (3)	N3—C15—C16	113.7 (3)
C13—N3—C15	125.2 (3)	N3—C15—H15A	108.8
C12—N3—C15	122.4 (3)	C16—C15—H15A	108.8
C18—N4—C22	113.0 (3)	N3—C15—H15B	108.8
C18—N4—C19	110.6 (3)	C16—C15—H15B	108.8
C22—N4—C19	108.9 (3)	H15A—C15—H15B	107.7
C18—N4—H4A	111 (2)	C15—C16—C17	114.7 (3)
C22—N4—H4A	106 (2)	C15—C16—H16A	108.6
C19—N4—H4A	107.1 (18)	C17—C16—H16A	108.6
C23—N5—C20	111.8 (3)	C15—C16—H16B	108.6
C23—N5—C21	112.6 (3)	C17—C16—H16B	108.6
C20—N5—C21	109.1 (3)	H16A—C16—H16B	107.6
C23—N5—H5A	107 (2)	C16—C17—C18	112.1 (3)
C20—N5—H5A	110 (2)	C16—C17—H17A	109.2
C21—N5—H5A	106 (2)	C18—C17—H17A	109.2
C2—C1—C6	120.6 (5)	C16—C17—H17B	109.2
C2—C1—H1	119.7	C18—C17—H17B	109.2
C6—C1—H1	119.7	H17A—C17—H17B	107.9
C3—C2—C1	120.3 (4)	N4—C18—C17	111.5 (3)
C3—C2—H2	119.9	N4—C18—H18A	109.3
C1—C2—H2	119.9	C17—C18—H18A	109.3
C2—C3—C4	120.3 (4)	N4—C18—H18B	109.3
C2—C3—Cl1	120.2 (4)	C17—C18—H18B	109.3
C4—C3—Cl1	119.5 (4)	H18A—C18—H18B	108.0
C3—C4—C5	119.5 (5)	C20—C19—N4	110.8 (3)
C3—C4—H4	120.3	C20—C19—H19A	109.5
C5—C4—H4	120.3	N4—C19—H19A	109.5
C4—C5—C6	121.2 (4)	C20—C19—H19B	109.5
C4—C5—H5	119.4	N4—C19—H19B	109.5
C6—C5—H5	119.4	H19A—C19—H19B	108.1
C5—C6—C1	118.2 (4)	N5—C20—C19	110.0 (3)
C5—C6—C7	120.8 (4)	N5—C20—H20A	109.7
C1—C6—C7	120.9 (4)	C19—C20—H20A	109.7
C8—C7—O1	109.0 (4)	N5—C20—H20B	109.7
C8—C7—C6	133.7 (4)	C19—C20—H20B	109.7
O1—C7—C6	117.2 (4)	H20A—C20—H20B	108.2
C7—C8—C9	107.7 (4)	N5—C21—C22	109.9 (3)
C7—C8—H8	126.1	N5—C21—H21A	109.7
C9—C8—H8	126.1	C22—C21—H21A	109.7
C10—C9—C8	106.7 (4)	N5—C21—H21B	109.7
C10—C9—H9	126.7	C22—C21—H21B	109.7
C8—C9—H9	126.7	H21A—C21—H21B	108.2
C9—C10—O1	110.0 (3)	C21—C22—N4	111.2 (3)
C9—C10—C11	131.0 (4)	C21—C22—H22A	109.4
O1—C10—C11	118.9 (4)	N4—C22—H22A	109.4
N1—C11—C10	121.3 (4)	C21—C22—H22B	109.4
N1—C11—H11	119.4	N4—C22—H22B	109.4
C10—C11—H11	119.4	H22A—C22—H22B	108.0
O2—C12—N2	128.1 (4)	N5—C23—H23A	109.5
O2—C12—N3	125.8 (4)	N5—C23—H23B	109.5
N2—C12—N3	106.1 (3)	H23A—C23—H23B	109.5
O3—C13—N3	124.9 (4)	N5—C23—H23C	109.5
O3—C13—C14	127.9 (4)	H23A—C23—H23C	109.5
N3—C13—C14	107.2 (3)	H23B—C23—H23C	109.5
N2—C14—C13	102.4 (3)	H4C—O4—H4B	108.2
			
C11—N1—N2—C12	−171.5 (4)	N1—N2—C12—N3	166.0 (3)
C11—N1—N2—C14	−7.7 (5)	C14—N2—C12—N3	−0.1 (4)
C6—C1—C2—C3	1.1 (7)	C13—N3—C12—O2	−179.5 (4)
C1—C2—C3—C4	−0.4 (7)	C15—N3—C12—O2	7.6 (6)
C1—C2—C3—Cl1	178.4 (3)	C13—N3—C12—N2	0.5 (4)
C2—C3—C4—C5	−0.6 (7)	C15—N3—C12—N2	−172.3 (3)
Cl1—C3—C4—C5	−179.4 (3)	C12—N3—C13—O3	−180.0 (4)
C3—C4—C5—C6	1.0 (7)	C15—N3—C13—O3	−7.4 (6)
C4—C5—C6—C1	−0.4 (6)	C12—N3—C13—C14	−0.7 (4)
C4—C5—C6—C7	177.3 (4)	C15—N3—C13—C14	171.9 (3)
C2—C1—C6—C5	−0.7 (6)	N1—N2—C14—C13	−165.1 (3)
C2—C1—C6—C7	−178.4 (4)	C12—N2—C14—C13	−0.3 (4)
C10—O1—C7—C8	0.1 (4)	N3—C13—C14—N2	0.6 (4)
C10—O1—C7—C6	−176.9 (3)	C13—N3—C15—C16	100.9 (4)
C5—C6—C7—C8	−165.5 (5)	C12—N3—C15—C16	−87.3 (4)
C1—C6—C7—C8	12.2 (7)	N3—C15—C16—C17	−61.7 (4)
C5—C6—C7—O1	10.5 (6)	C15—C16—C17—C18	−66.6 (4)
C1—C6—C7—O1	−171.9 (4)	C22—N4—C18—C17	−70.6 (4)
O1—C7—C8—C9	−0.4 (5)	C19—N4—C18—C17	167.0 (3)
C6—C7—C8—C9	175.8 (4)	C16—C17—C18—N4	−173.7 (3)
C7—C8—C9—C10	0.6 (5)	C18—N4—C19—C20	−178.4 (3)
C8—C9—C10—O1	−0.6 (5)	C22—N4—C19—C20	56.9 (4)
C8—C9—C10—C11	175.8 (4)	C23—N5—C20—C19	−174.5 (3)
C7—O1—C10—C9	0.3 (4)	C21—N5—C20—C19	60.4 (4)
C7—O1—C10—C11	−176.6 (3)	N4—C19—C20—N5	−59.7 (4)
N2—N1—C11—C10	177.1 (3)	C23—N5—C21—C22	175.5 (3)
C9—C10—C11—N1	−170.6 (4)	C20—N5—C21—C22	−59.8 (4)
O1—C10—C11—N1	5.5 (6)	N5—C21—C22—N4	58.8 (4)
N1—N2—C12—O2	−13.9 (6)	C18—N4—C22—C21	−179.9 (3)
C14—N2—C12—O2	179.9 (4)	C19—N4—C22—C21	−56.6 (4)

### Hydrogen-bond geometry (Å, °)

**Table:**

*D*—H···*A*	*D*—H	H···*A*	*D*···*A*	*D*—H···*A*
O4—H4B···Cl3	0.86	2.40	3.250 (3)	169
O4—H4C···Cl3^i^	0.85	2.50	3.250 (3)	148
N5—H5A···Cl2^ii^	1.05 (4)	1.95 (4)	2.995 (3)	174 (3)
N4—H4A···Cl3	1.04 (4)	2.00 (4)	3.035 (3)	178 (3)

Symmetry codes: (i) −*x*, *y*, −*z*+1/2; (ii) *x*, −*y*+2, *z*+1/2.
